# Supplementary material for: Integrin-dependent migratory switches regulate the translocation of Toxoplasma-infected dendritic cells across brain endothelial monolayers
Source: Cell Mol Life Sci. 2021 May 22;78(12):5197–212. doi: 10.1007/s00018-021-03858-y (PMC8254729; doi:10.1007/s00018-021-03858-y)
Supplement: Supplementary file 5 — Supplementary file5 (DOCX 16 KB) Supplementary Table S1. Sequences for shRNAs. Supplementary Table S2. Sequences for qPCR primers. [file 18_2021_3858_MOESM5_ESM.docx]

**Table S1. Sequences for shRNAs.**

| **Target** | **Sequence (5’ to 3’)** | |
| --- | --- | --- |
| shLuc: | | TGTTCTCCGAACGTGTCACGTTTCAAGAGAACGTGACACGTTCGGAGAACTTTTTTC |
|  |  |  |
| shITGB1: | | CCGGGCCATTACTATGATTATCCTTCTCGAGAAGGATAATCATAGTAATGGCTTTTTG |
|  |  |  |
| shTln1: | | TGCTGGGAAAGCTTTGGACTACTACTTCAAGAGAGTAGTAGTCCAAAGCTTTCCCAGCTTTTTTC |

|  |
| --- |

**Table S2. Sequences for qPCR primers.**

| **Target** | **Primer pair sequence (5’ to 3’)** |
| --- | --- |
| *Tln1* | Fd: GGTGAAGACTATCATGGTGG  Rv: TTGGTGATACCAATTCGGG |
| *Itgb1* | Fd: GATGAATTTGCAACTGGTTTCC  Rv: GCAAGATTTGGCATTTGCT |
| *Gapdh* | Fd: TGACCTCAACTACATGGTCTACA  Rv: CTTCCCATTCTCGGCCTTG |
| *Vcam1* | Fd: GTGACTCCATGGCCCTCACTT  Rv: CGTCCTCACCTTCGCGTTTA |
| *Icam1* | Fd: CAATTTCTCATGCCGCACAG  Rv: CTGGAAGATCGAAAGTCCGG |
| *Sele (E-selectin)* | Fd: CCCTGCCCACGGTATCAG  Rv: ACGTGCATGTCGTGTTCCA |
| *Itga4* | Fd: ATGGCTGCGGAAGCGATGTGC  Rv: CATGCCATAGCAAACACCAGTGG |
| *Itgb2* | Fd: CAGGAATGCACCAAGTACAAAGT  Rv: CCTGGTCCAGTGAAGTTCAGC |
| *CD31* | Fd: CTGCCAGTCCGAAAATGGAAC  Rv: CTTCATCCACCGGGGCTATC |
| *Cldn5* | Fd: TTTCTTCTATGCGCAGTTGG  Rv: GCAGTTTGGTGCCTACTTCA |
| *Itgax* | Fd: CTTCCCAGACTTGAAGACC  Rv: TCTTCTCCATCATTAGACACC |
